# Physical modeling and geometric shape simulation for one-dimensional flexible objects with cylindrical surface constraints

**DOI:** 10.1038/s41598-023-32064-y

**Published:** 2023-03-24

**Authors:** Yuhang Mei, Hongwang Du, Qinwen Jiang, Wei Xiong

**Affiliations:** Ship Electromechanical Equipment Institute, Room 315, Mechanical and Electrical Building, Dalian Maritime University, No.1 Linghai Road, Ganjingzi District, Dalian, 116026 Liaoning China

**Keywords:** Mechanical engineering, Applied mathematics, Nonlinear phenomena

## Abstract

This study develops forces equilibrium differential equations for the geometric modeling of 1D flexible objects with surface constraints. These second-order equations are an extension of the Cosserat elastic rod theory and include both bending and torsion. Variables were established for the centerline and attitude in the Cartesian coordinate system of the cross section. This paper specifically investigates the case of a 1D flexible object constrained by a cylindrical surface. To solve this problem, a novel hybrid semi-analytical numerical method is proposed. In this process, a Hamiltonian function and an initial integral operator are introduced in a cylindrical coordinate system. The analytical solution, decoupled in polar coordinates, is then derived. The improved finite difference method was then used to obtain three cylindrical coordinates, which ensured numerical stability and efficiency. The results of a geometric shape simulation with differing boundary conditions demonstrate that this proposed method is capable of real-time modeling. As such, this technique could be a promising new tool for use in graphics simulations of elongated structures, such as DNA molecules, drill pipes, and submarine cables.

## Introduction

The real-time simulation of complex and increasingly realistic objects has become an active area of research in recent decades, driven by the continuous development of virtual reality technology and computer graphics capabilities^1^. Recent object simulation directions have primarily focused on two fields: the study of rigid objects and the study of flexible objects^2^. The study of rigid object has seen the development of increasingly mature theories, while the study of flexible objects remains in its early stages^3^. The rapid development of flexible materials has created a need for more advanced simulation models^4^.

When a rigid object is subjected to forces, its shape can be regarded as constant on a macroscopic scale. In contrast, flexibility refers to the deformation of objects on this same scale, when subjected to a force^5^. The difficulty in simulating flexible objects lies in modeling the complex mechanisms of the deformation process^6^. Flexible objects can be divided into one-dimensional (1D), two-dimensional (2D), and three-dimensional (3D) according to their physical form^7^. One-dimensional flexible objects (1DFOs) are widely used to model drill pipes, molecular DNA chains, wires, hair, cables, ropes, branches, etc. Two-dimensional flexible objects include cloth, shells, etc. Three-dimensional flexible objects have been used to model humans, animals, and spheres^8–10^. This study focuses specifically on the modeling and simulation of 1DFOs with provided surface constraints.

At present, three primary methods are used to simulate 1DFOs: geometric, physical, and hybrid models^11^. The simulation of 1DFOs using geometric models is conducted by describing and expressing the body. This description only considers geometric information (the shape, position, and size of an object in Euclidean space) and topological information (the number of topological elements and the connections between them)^12^. In addition to representing the external characteristics of a flexible body, physical 1DFO models can analyze internal structure and provide a more realistic simulation effect^13,14^. Since the deformation of 1DFO conforms to the deformation characteristics of slender structure, the physical model of 1DFO based on elasticity can obtain the real shape of 1DFO in equilibrium state by analyzing the potential energy function and boundary conditions of 1DFO^42,43^. As such, the physical model based on elasticity theory has become a common approach in recent years.

## Related work

1DFOs are slender by definition, with a length much larger than their cross-section. Mechanical modeling techniques for 1DFOs include the energy curve method^15^, the finite element method^16^, multi-body dynamics^17^, the mass-spring method^18^, and the non-linear mechanics of elastic rods^19^.

The energy curve method considers 1DFOs to be splines with stored energy, modeled by Bezier, B-curve, and NURBS splines. 1DFOs consist of geometric curve segments that are manipulated by a series of control points, which represent the degrees of freedom within a system, rather than locations of matter, gravity, or viscous force. The static equilibrium of 1DFOs satisfies the principle of minimum potential energy and the dynamic problem satisfies the Lagrangian dynamics equation. With this approach, Rémion et al. studied shape simulations for a rope, including the cases of knotting and winding^15^. Baaser et al. described a brake hose using a spline curve. This was done by calculating the total energy of the hose and optimizing its length^20^. Valentini et al. studied cables in an augmented reality environment using this model^21^. Menon et al. studied the motion simulation and rendering of 1DFOs^22^. However, the deformation relation of 1DFOs can only be approximated by this method, but the accuracy cannot be guaranteed, and the torsional deformation is well described. Therefore, it is not suitable for precise modeling of 1DFOs.

The finite element method has been used in previous studies to discretize 1DFOs into discrete polyhedron meshes. The deformation and stress in each mesh can then be described by an interpolating function, from which the shape of the 1DFO can be obtained^16^. Conventional finite element analysis software, such as ANSYS or ABAQUS, uses this approach to model the deformation of 1DFOs. Martin et al. proposed a joint finite element simulation method using rods and shells, in which an embedded triangular mesh was used to express geometric shapes. A meshless Hermite interpolation method was then proposed to ensure efficient and accurate simulations that agreed with the theory of elasticity. This approach avoided the need to distinguish dimensions for flexible bodies^23^. Zhao et al. used a similar method to analyze tree morphology, with each frame processing hundreds of branches and more than 100,000 leaves^24^. Although this method has high computational accuracy, it requires a large amount of computation, which affects the quick solution of 1DFOs shape.

Multi-body dynamics methods first divide 1DFOs into a combination of connected rods and masses, which are then modeled using analytical mechanics^17^. Servin et al. used multi-body dynamics to simulate the cable hoisting system of a crane in a virtual environment. The integral time step reached 1/60 s, which ensured high numerical stability^25^. Ham et al. studied a restrained wire rope on a crane^26^ and Andrews et al. conducted a similar study^27^. Although this method has better solving efficiency, torsion and external forces are not taken into account. Therefore, it is not suitable for accurate modeling of 1DFOs.

The mass-spring method is currently the most widely used technique in 1DFO modeling. In this approach, 1DFOs are connected by a series of massless particles and a series of massless, non-zero-length springs. Linear springs between adjacent particles describe tension in the 1DFO. Bending springs and torsion springs are further introduced between adjacent springs to describe bending and torsional deformations^18^. This mass-spring method is simple to establish and is widely used in hair simulations^18^, surgery wire modeling^28^, and cable assembly simulations^29^. This modeling method has better solving efficiency and real-time performance. However, this method cannot describe the influence of contact forces, so it is not suitable for the modeling of 1DFOs constrained by cylinder.

Nonlinear elastic rod mechanics (in which a rigid cross section is assumed) were first used to analyze the mechanical properties of DNA. The spatial geometry of 1DFOs can be described by the displacement and rotation of such a cross section relative to a centerline^19^. Pai et al. applied this theory to the modeling of sutures^19^. Hu et al. used this same theory to model hair in animated video games^30^. Tang et al. modeled guide wires in virtual surgery simulations^31^. Hermansson et al. simulated cable geometry based on the Cosserat theory^32^. This modeling method can consider torsion and has good accuracy. Among these techniques, nonlinear elastic rod mechanics are perhaps the most effective for modeling and simulating 1DFOs. Therefore, the elastic rod modeling theory is chosen in this paper.

Though there are many studies utilizing this approach, current research primarily focuses on the simple case of a 1DFO, which is often fixed at one or both ends. These studies rarely consider the complex constraints that exist in real systems, specifically: contact friction and collision contact while in motion. Bergou et al. used Cosserat theory to study the closed-loop and winding phenomena of cables due to self-contact^33^. Spillmann et al. described friction torque between the hand and a 1DFO using Lagrangian dynamics^34^. Wang et al. conducted a puncture simulation for surgery wires, considering impact contact and friction^35^. Similarly, Chai et al. studied the simulation of contact deformation between hair bundles and other rigid objects^36^. Most of the contact types considered in the above literature are point contact or line contact, while there are few studies on the deformation of 1DFOs during surface contact. Therefore, this paper aims to study the morphological changes of 1DFOs under curved surface contact.

As such, nonlinear elastic rod mechanics are the most robust choice for modeling and simulating 1DFOs. However, to date, only friction and collision contact forces have been considered in the literature. In practical applications, surface constraints are of critical importance and can directly affect the behavior of an object. For example, DNA molecules coiled around proteins, drill pipes with borehole walls, and submarine cables are all subject to surface constraints. As such, accurately simulating 1DFOs constrained by surfaces in virtual environments is of significant importance in the field. This paper presents a novel methodology for modeling and simulating such constraints using the Cosserat elastic rod theory. The main contributions of this paper are as follows:

1. Aiming at the problem of 1DFOs constrained by cylindrical surface, the modeling of 1DFOs constrained by curved surface is completed in this paper by combining Cosserat elastic bar theory.
2. Based on the above model, a hybrid semi-analytic numerical calculation method is proposed to solve the morphology of 1DFOs constrained by surface based on the difference method.

Section "Related work" introduces the research status and shortcomings of 1DFOs modeling. Section "Modeling of 1DFOs with surface constraints" introduces 1DFOs modeling and constraints. Section "Application: equilibrium problem and numerical simulation on a cylindrical surface" introduces the numerical solution process. In "Solution and results" section, the solution algorithm is designed and the results are analyzed. The conclusion is given in "Conclusion" section.

## Modeling of 1DFOs with surface constraints

### Geometric shape descriptions

**Figure 1:**
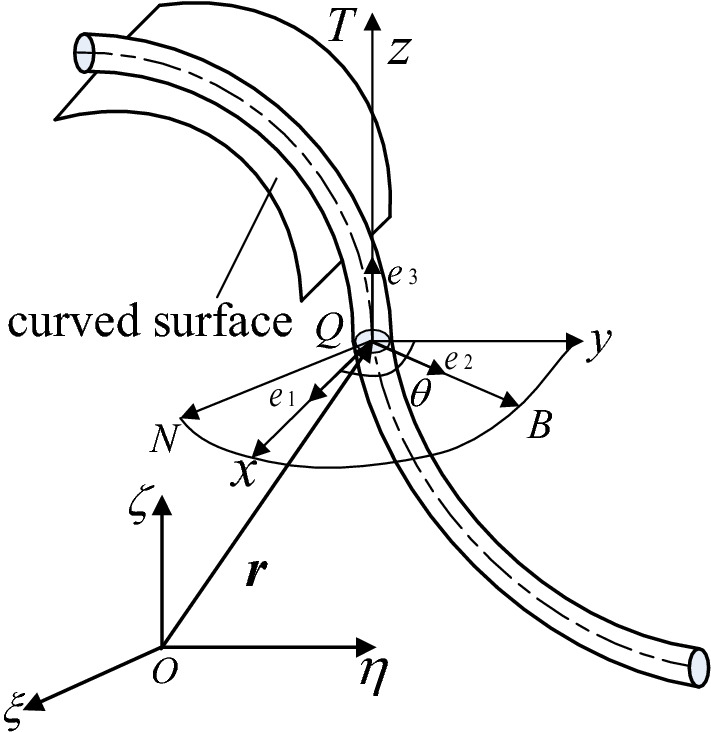
A 1DFO subject to surface constraints.

A 1DFO of length L, with a circular cross-section subject to surface constraints, was used as the research object in this study (see Fig. 1).

A fixed coordinate system *O*-*ξηζ* was established with three unit basis vectors: **e**_ζ_, **e**_η_, and **e**_ξ_. The vector diameter of the 1DFO centerline in this fixed system can be expressed as:

1 $${\mathbf{r}}\left( s \right) = \xi \left( s \right){\mathbf{e}}_{{\upxi }} + \eta \left( s \right){\mathbf{e}}_{{\upeta }} + \zeta \left( s \right){\mathbf{e}}_{{\upzeta }}$$

where *s* represents the arc coordinates. Assuming the 1DFO to be unstretched requires:

2 $$\left\| {{\dot{\mathbf{r}}}} \right\| = 1.$$

A centroid principal axis coordinate system (*p*-*xyz*) can then be established for any point *Q* on the centerline, connected with 1DFO sections. This results in a Frenet coordinate system (*p*-*NBT*). According to the Angle θ between the two coordinate systems, the torsion between the section of 1DFO is expressed. And, the unit base vectors of the *p*-*xyz* system are ***e***_1_, ***e***_2_, and ***e***_3_, respectively. The vector ***e***_3_ is then tangent to the centerline:

3 $${\dot{\mathbf{r}}} = {\mathbf{e}}_{3} ,$$

pointing in the direction of increasing arc coordinates.

A 1DFO segment can then assume two different orientations within these arc coordinate *s*. The cross-section whose normal vector points in the direction of increasing arc coordinates is called the positive cross-section (denoted as s^+^). Conversely, the negative cross-section (whose normal points in the direction of decreasing arc coordinates) is denoted as s^-^. The shape of the 1DFO is then a realization of the displacement and rotation of *p*-*xyz* along the center relative to *O*-*ξηζ*. The angular velocity of the cross-section relative to *O*-*ξηζ* is defined as the degree of bending and torsion, denoted as **ω**:

4 $${\dot{\mathbf{e}}} = {{\varvec{\upomega}}} \times {\mathbf{e}}.$$

In this system^40^, **e** is a fixed vector in *O*-*ξηζ*. Therefore, **ω** can be expressed by the unit vectors ***e***_1_, ***e***_2_, and ***e***_3_ as:

5 $${{\varvec{\upomega}}} = \left( {{\dot{\mathbf{e}}}_{2} \cdot {\mathbf{e}}_{3} } \right){\mathbf{e}}_{1} + \left( {{\dot{\mathbf{e}}}_{3} \cdot {\mathbf{e}}_{1} } \right){\mathbf{e}}_{2} + \left( {{\dot{\mathbf{e}}}_{1} \cdot {\mathbf{e}}_{2} } \right){\mathbf{e}}_{3}$$

### Surface constraint representation

In the fixed coordinate system (*O*-*ξηζ*), 1DFO surface constraints can be described as:

6 $$g\left( {\xi ,\eta ,\zeta } \right) = 0.$$

The surface of a constrained 1DFO is assumed to be continuous, smooth, and bilateral. This implies that only a single point contacts the surface on the boundary circumference of the cross-section. In other words, the coordinates of this point satisfy Eq. (6). The 1DFO must remain on one side of the constraint surface and is excluded from passing through or leaving this surface. Friction between the 1DFO and the constraint surface was neglected and the cross-section was considered to be rigid (without deformations). These surface constraints naturally impose spatial position restrictions on the 1DFO. In the absence of surface constraints, the centerline plays a key role in modeling deformations. It is therefore necessary to find a basic relationship between the position of the centerline and the surface constraint Eq. (6), yielding a constraint equation for the centerline.

When a 1DFO is constrained by a surface, points on this surface can be expressed as:

7 $${{\varvec{\rho}}} = \left( {\begin{array}{*{20}l} {\xi_{c} } & {\eta_{c} } & {\zeta_{c} } \\ \end{array} } \right)\left( {\begin{array}{*{20}l} {{\varvec{e}}_{\xi } } \\ {{\varvec{e}}_{\eta } } \\ {{\varvec{e}}_{\zeta } } \\ \end{array} } \right)$$

and are assumed to contact the cross-section boundary of the centerline sagittal radius **r**, which satisfies:

8 $${\varvec{r}} = {{\varvec{\rho}}} + a{\varvec{n}}_{c} .$$

Here, a is the sectional radius of the 1DFO and ***n***_c_ is the unit normal vector for the constrained surface at point ***ρ***. According to Eq. (8), ***ρ*** could be expressed in the Cartesian coordinates of the 1DFO centerline as:

9 $$\begin{array}{*{20}l} {\xi_{c} = \xi_{c} \left( {\xi ,\eta ,\zeta ,an_{c\xi } } \right),} \\ {\eta_{c} = \eta_{c} \left( {\xi ,\eta ,\zeta ,an_{c\eta } } \right),} \\ {\zeta_{c} = \zeta_{c} \left( {\xi ,\eta ,\zeta ,an_{c\zeta } } \right)} \\ \end{array}$$

where *n*_*cξ*_*, n*_*cη*_*,* and *n*_*cζ*_ are the projection of ***n***_c_ in the fixed system *O*-*ξηζ*. Equation (6) can then be used to generate an expression for the 1DFO with surface constraints:

10 $$g\left( {\begin{array}{*{20}l} {\xi_{c} \left( {\xi ,\eta ,\zeta ,an_{c\xi } } \right),\eta_{c} \left( {\xi ,\eta ,\zeta ,an_{c\eta } } \right)} \hfill \\ {,\zeta_{c} \left( {\xi ,\eta ,\zeta ,an_{c\zeta } } \right)} \hfill \\ \end{array} } \right) = 0.$$

In addition, Eq. (8) requires the following:

11 $${\text{e}}_{{3}} \cdot n_{c} = 0.$$

This expression implies that when a 1DFO is constrained by a surface, its cross-section must be perpendicular to the normal vector of the constraint surface at that contact point. Equations (10) and (11) constitute a ‘constant geometric constraint’ on the 1DFO cross-section. Four coordinates are then needed to determine the position and orientation of the cross-section constrained by the surface.

The distribution of force ***f*** along the contact line of the constraint surface could then be determined as:

12 $$\begin{aligned} {\varvec{f}} &= \lambda \cdot n_{c} , \hfill \\ {\varvec{n}}_{{\varvec{c}}} &= \frac{\partial g}{{\partial \xi_{c} }}{\text{e}}_{{\upxi }} + \frac{\partial g}{{\partial \eta_{c} }}{\text{e}}_{{\upeta }} + \frac{\partial g}{{\partial \zeta_{c} }}{\text{e}}_{{\upzeta }} . \hfill \\ \end{aligned}$$

Here, λ is a Lagrange multiplier which assumes the 1DFO to be on the same side of the normal vector ***n***_c_.

### Euler angle description

**Figure 2:**
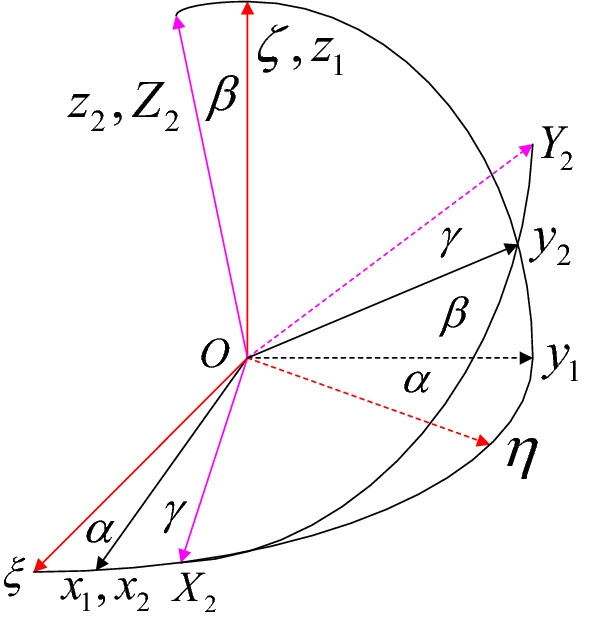
The Eulerian angles.

The cross-sectional attitude of the 1DFO can be represented by the three Euler angles α (s), β (s), and γ (s). These describe the space attitude coordinates of the *p*-*xyz* system relative to the *O*-*ξηζ* system, as shown in Fig. 2.

The directional cosine transformation matrix between the *p*-*xyz* system and the *O*-*ξηζ* system can be represented as:

13 $$\begin{array}{*{20}l} {\left( {\begin{array}{*{20}l} {{\mathbf{e}}_{\xi } } \\ {{\mathbf{e}}_{\eta } } \\ {{\mathbf{e}}_{\zeta } } \\ \end{array} } \right) = {\mathbf{T}} \times \left( {\begin{array}{*{20}l} {{\mathbf{e}}_{1} } \\ {{\mathbf{e}}_{2} } \\ {{\mathbf{e}}_{3} } \\ \end{array} } \right)} \hfill \\ {{\mathbf{T}} = \left( {\begin{array}{*{20}l} {{\text{c}}\alpha {\text{c}}\gamma - {\text{c}}\beta {\text{s}}\alpha {\text{s}}\gamma } & { - {\text{c}}\alpha {\text{s}}\gamma - {\text{c}}\beta {\text{s}}\alpha {\text{c}}\gamma } & {{\text{s}}\beta {\text{s}}\alpha } \\ {{\text{s}}\alpha {\text{c}}\gamma + {\text{c}}\beta {\text{c}}\alpha {\text{s}}\gamma } & { - {\text{s}}\alpha {\text{s}}\gamma - {\text{c}}\beta {\text{c}}\alpha {\text{c}}\gamma } & { - {\text{s}}\beta {\text{c}}\alpha } \\ {{\text{s}}\beta {\text{s}}\gamma } & {{\text{s}}\beta {\text{c}}\gamma } & {{\text{c}}\beta } \\ \end{array} } \right)} \hfill \\ \end{array} ,$$

where *c* and *s* represent cosine and sine operators, respectively^37^. The Euler angle expression of angular velocity can be derived by combining Eqs. (5) and (13):

14 $$\left( {\begin{array}{*{20}c} {\omega_{1} } \\ {\omega_{2} } \\ {\omega_{3} } \\ \end{array} } \right) = \left( {\begin{array}{*{20}c} {\frac{{{\text{d}}\alpha }}{{{\text{d}}s}} + \frac{{{\text{d}}\beta }}{{{\text{d}}s}}\cos \gamma } \\ {\frac{{{\text{d}}\alpha }}{{{\text{d}}s}}\sin \beta \cos \gamma - \frac{{{\text{d}}\beta }}{{{\text{d}}s}}\sin \gamma } \\ {\frac{{{\text{d}}\alpha }}{{{\text{d}}s}}\cos \beta + \frac{{{\text{d}}\gamma }}{{{\text{d}}s}}} \\ \end{array} } \right)$$

The relationship between Cartesian coordinates (*ξ, η, ζ*) and the Euler angles for the 1DFO centerline can be derived by combining Eqs. (3) and (13):

15 $$\left\{ {\begin{array}{*{20}l} {\frac{{{\text{d}}\xi }}{{{\text{d}}s}} = \sin \beta \sin \alpha } \\ {\frac{{{\text{d}}\eta }}{{{\text{d}}s}} = - \sin \beta \cos \alpha } \\ {\frac{{{\text{d}}\zeta }}{{{\text{d}}s}} = \cos \beta .} \\ \end{array} } \right..$$

The surface constraint distribution force can then be written as a component in the principal axis coordinate system represented by the Euler angles:

16 $${\varvec{f}} = \lambda \left( {\begin{array}{*{20}l} {\frac{\partial g}{{\partial \xi_{c} }}} & {\frac{\partial g}{{\partial \eta_{c} }}} & {\frac{\partial g}{{\partial \zeta_{c} }}} \\ \end{array} } \right) \times {\varvec{T}} \times \left( {\begin{array}{*{20}l} {{\varvec{e}}_{1} } \\ {{\varvec{e}}_{2} } \\ {{\varvec{e}}_{3} } \\ \end{array} } \right).$$

This expression satisfies ***f***·***e***_3_ = 0. If a constraint is one-sided, the 1DFO can be either on or off the constraint surface but cannot enter or cross over the constraint surface. Therefore, the constraints on the 1DFO in the arc coordinates *s* can be expressed as:

17 $$\lambda = {\varvec{f}} \cdot {\varvec{n}}_{{\varvec{c}}} \ge 0.$$

### Equilibrium equations

The objects of interest in this section are assumed to be 1DFO element segments with arc coordinates *s* and *s* + ∆*s* on the surface. Force equilibrium differential equations can be established in the case of ∆*s* → 0:

18 $$\left\{ \begin{aligned} &\frac{{{{\text{d}}}{\mathbf{F}}}}{{{\text{d}}s}} + {{\varvec{\upomega}}} \times {\mathbf{F}} + {\mathbf{f}} = 0 \hfill \\ &\frac{{{{\text{d}}}{\mathbf{M}}}}{{{\text{d}}s}} + {{\varvec{\upomega}}} \times {\mathbf{M}} + {\mathbf{e}}_{3} \times {\mathbf{F}} = 0 \hfill \\ \end{aligned} \right..$$

Here, ***F*** and ***M*** are the principal vector and moment of internal force at the center of a shape on the s^+^ cross-section, ‘~’ represents a derivative in the *p*-*xyz* system, and ***f*** is the line distribution constraint force on the 1DFO surface.

By considering the original curvature and torsion of the 1DFO, the cross-section moment can be expressed as:

19 $$\begin{array}{*{20}l} {M_{1} = A\left( {\omega_{1} - \omega_{1}^{0} } \right),} \\ {M_{2} = B\left( {\omega_{2} - \omega_{2}^{0} } \right),} \\ {M_{3} = C\left( {\omega_{3} - \omega_{3}^{0} } \right)} \\ \end{array} ,$$

using material mechanics. Here, A and B are the bending stiffness of the cross-section around the *x*-axis and *y*-axis, respectively. C is the torsional stiffness of the cross-section around the *z*-axis. The stiffness coefficient is constant for an isotropic flexible object with a circular cross-section. This value is determined by the material coefficient and the 1DFO cross-section shape:

20 $$A = B = \frac{\pi }{16}Ea^{4} ,C = \left( {\frac{1}{1 + \nu }} \right)\frac{\pi }{16}Ea^{4} ,$$

where *E* is Young’s modulus and *ν* is the Poisson ratio. The values assumed in this study are listed in Table 1.

**Table 1:** Simulation parameters.

Material Parameters	E (N/cm^2^)	1.07 × 10^4^
	ν	0.25
	R (cm)	0.75
	a (cm)	0.25
Euler Angles and Derivatives	$\alpha_{0}$ (rad)	0
	$\beta_{0}$ (rad)	π/6
	$\gamma_{0}$ (rad)	− 0.1
	$\beta_{0}^{\prime }$	0.5
	$\gamma_{0}^{\prime }$	0.5
Boundary Conditions	$F_{10}$ (N)	5
	$F_{30}$ (N)	10

In summary, the 1DFO system subject to surface constraints consists of a static equilibrium Eq. (18), a linear constitutive Eq. (19), deformed geometric Eqs. (14, 15), constraint Eqs. (10, 11), and a constraint expression (16). These equations are closed and can be solved if the corresponding initial/boundary conditions are given. The geometric shape of the 1DFO constrained by a surface can then be determined.

## Application: equilibrium problem and numerical simulation on a cylindrical surface

**Figure 3:**
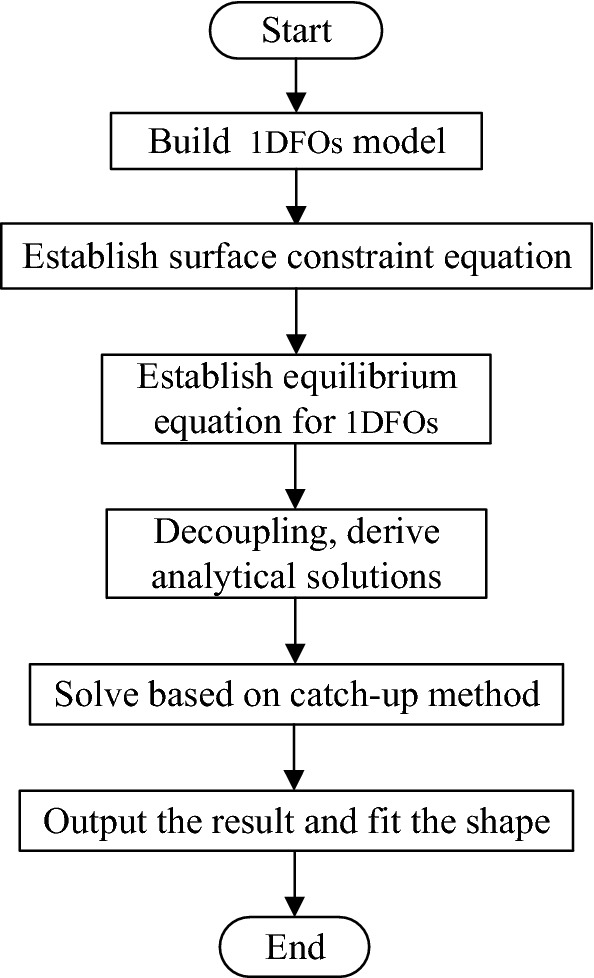
A 1DFO shape solving procedure.

In this paper, the modeling and solving process of 1DFO constrained by cylinder is shown in Fig. 3. In "Modeling of 1DFOs with surface constraints" section, the equilibrium equation and constraint equation of 1DFO are established. In this section, the derivation of the analytical formula for 1DFO constrained by cylinders will be introduced.

### Modeling of a 1DFO on a cylindrical surface

**Figure 4:**
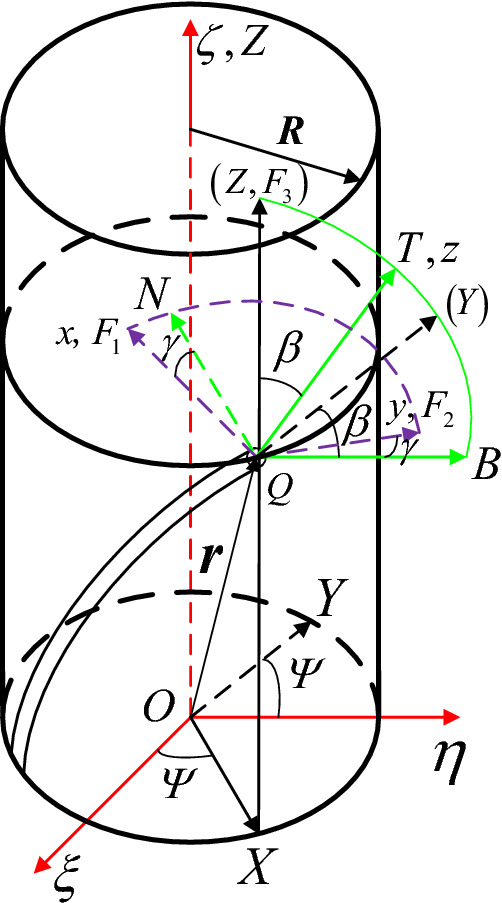
A 1DFO under cylindrical surface constraints.

A circular 1DFO cross-section under cylindrical surface constraints is shown in Fig. 4.

Cylindrical coordinates were used to represent the 1DFO geometry and constraints. Ignoring the original curvature and torsion, the 1DFO was constrained by a cylindrical surface of radius *R*. A fixed coordinate system was then established in which the center of the bottom of the cylinder served as the origin and the central cylinder axis formed the *ζ* axis. The *O-ξηζ* coordinates can be transformed into the cylindrical coordinate system *O*-*XYZ* with a rotation through the angle *α*. The three cylindrical coordinates (*ρ*, *α*,* ζ*) can then describe the geometry of the 1DFO. Any point *Q* on the centerline is constrained by the cylindrical surface in the *XOZ* coordinate plane, where the X and Z axes point in the radial and circumferential directions, respectively.

The distance between the point *Q* and the *Z* axis is then:

21 $$\rho = R + a.$$

Two additional coordinate systems must then be established to describe the spatial position of any point *Q* on the centerline, relative to the fixed coordinate system. *P*-*NBT* was obtained from the cylindrical coordinate system *O*-*XYZ* by a rotation around the negative *X*-axis, through an angle *β*. The term *N* represents the negative direction of the *X*-axis, *T* is the tangent direction of the point *Q* on the centerline, and *B* is the sub-normal direction. *Q*-*NBT* is the Frenet coordinate system for the 1DFO constrained by a cylindrical surface.

The relationship between the fixed system *O-ξηζ* and the cylindrical system *O*-*XYZ* is as follows:

22 $$\left\{ \begin{aligned} &\frac{{{\text{d}}\rho }}{{{\text{d}}s}} = \frac{1}{\rho }\left( {\xi \frac{{{\text{d}}\xi }}{{{\text{ds}}}} + \eta \frac{{{\text{d}}\eta }}{{{\text{ds}}}}} \right) \hfill \\ &\frac{{{\text{d}}\alpha }}{{{\text{d}}s}} = \frac{1}{{\rho^{2} }}\left( {\xi \frac{{{\text{d}}\eta }}{{{\text{ds}}}} - \eta \frac{{{\text{d}}\xi }}{{{\text{ds}}}}} \right) \hfill \\ \end{aligned} \right..$$

The geometric conditions satisfied by the cylindrical constraints can be determined by combining Eqs. (3), (13), and (15):

23 $$\frac{{{\text{d}}\alpha }}{{{\text{d}}s}} = \frac{1}{\rho }\sin \beta = \frac{1}{R + a}\sin \beta .$$

By replacing Eqs. (10) and (11) with (23), while substituting (14), (15), (16), and (19) into the force equilibrium differential equations, an algebraic system in the form of Euler angles can be acquired for the 1DFO subject to surface constraints:

24 $$\left\{ \begin{aligned} &\frac{{{\text{d}}F_{1} }}{{{\text{d}}s}} + \frac{{{\text{d}}\alpha }}{{{\text{d}}s}}(F_{3} sin\beta - F_{2} cos\beta ) - f = 0 \hfill \\ &\frac{{{\text{d}}F_{2} }}{{{\text{d}}s}} + \frac{{{\text{d}}\alpha }}{{{\text{d}}s}}F_{1} cos\beta - \frac{{{\text{d}}\beta }}{{{\text{d}}s}}F_{3} = 0 \hfill \\ &\frac{{{\text{d}}F_{3} }}{{{\text{d}}s}} - \frac{{{\text{d}}\alpha }}{{{\text{d}}s}}F_{1} sin\beta + \frac{{{\text{d}}\beta }}{{{\text{d}}s}}F_{2} = 0 \hfill \\ &\frac{{{\text{d}}^{2} \beta }}{{{\text{d}}s^{2} }} - \left( {\frac{{{\text{d}}\alpha }}{{{\text{d}}s}}} \right)^{2} cos\beta sin\beta + \hfill \\ &\frac{{M_{3} }}{A}\frac{{{\text{d}}\alpha }}{{{\text{d}}s}}sin\beta - \frac{{F_{2} }}{A} = 0 \hfill \\ &\frac{{{\text{d}}^{2} \alpha }}{{{\text{d}}s^{2} }} + 2\left( {\frac{{{\text{d}}\alpha }}{{{\text{d}}s}}} \right)\left( {\frac{{{\text{d}}\beta }}{{{\text{d}}s}}} \right)cos\beta - \hfill \\ &\frac{{M_{3} }}{A}\frac{{{\text{d}}\beta }}{{{\text{d}}s}} + \frac{{F_{1} }}{A} = 0 \hfill \\ &\frac{{{\text{d}}\gamma }}{{{\text{d}}s}} + \frac{{{\text{d}}\alpha }}{{{\text{d}}s}}cos\beta = \frac{{M_{3} }}{C}. \hfill \\ \end{aligned} \right.$$

Here, *M*_3_ is the projection of the primary moment vector on the tangent axis of the 1DFO, which is constant for a circular cross-section. In summary, seven equations (consisting of (23) and (24)) have been developed to determine seven unknown variables *F*_*i*_, (α, β, γ), and *f*. Assuming the Euler angles can be determined in these coordinates, the geometric 1DFO shape (subject to cylindrical constraints) can then be acquired.

### Initial integration of the 1DFO model

Although this differential equation system (24) is closed, there is no analytic solution. The second-order nonlinearity of this system makes it difficult to guarantee stability and efficiency when directly applying numerical techniques such as Euler's method. To solve this problem, a novel hybrid semi-analytical numerical method is presented in this paper. A Hamiltonian function and initial integral operator are introduced in cylindrical coordinates to derive an analytical solution in decoupled polar coordinates. These polar coordinates can then be solved by a generic numerical integration algorithm, which can ensure high accuracy and efficiency.

The equilibrium equations for a constrained 1DFO are similar to that of an unconstrained system, but with the addition of a normal contact force. The projection of cross-section forces in cylindrical coordinates (*O*-*XYZ*) is as follows:

25 $$\left( {\begin{array}{*{20}l} {F_{X} } \\ {F_{Y} } \\ {F_{Z} } \\ \end{array} } \right) = \left( {\begin{array}{*{20}c} { - F_{1} } \\ { - F_{2} \cos \beta + F_{3} \sin \beta } \\ {F_{2} \sin \beta + F_{3} \cos \beta } \\ \end{array} } \right).$$

As the constraint force is orthogonal to the *Z*-axis, the normal contact force does not affect the projection conservation of the cross-section force along the *Z*-axis. The conservative value, the component of the pulling force acting on both ends of the 1DFO along the *Z*-axis, is represented by *F*_*Z*0_. This result can also be obtained by adding the second (multiplying by sinβ) and third (multiplying by cosβ) rows in Eq. (24) as follows:

26 $$F_{Z} = F_{Z0} .$$

*F*_1_ and *F*_2_ can be determined using the fifth and sixth rows in Eq. (24). The following expression can then be obtained by integrating the third row in Eq. (24):

27 $$\left( {\frac{{{\text{d}}\beta }}{{{\text{d}}s}}} \right)^{2} + \left( {\frac{{{\text{d}}\alpha }}{{{\text{d}}s}}} \right)^{2} \sin^{2} \beta + \frac{{2F_{3} }}{A} = h.$$

This expression implies that the sum of tangential projections for strain energy density and cylindrical restraint forces are conserved along the arc coordinates. This is because the contact force is normal, which means the force component of the moment along the tangent is not affected. The term *ω*_3_ = *ω*_30_ is the constant torsion rate for the 1DFO with a circular cross-section:

28 $$\left\{ \begin{array}{l} h = \frac{2H}{A} - \frac{C}{A}\omega_{30} \hfill \\ H_{0} = \frac{1}{2}\left( {\frac{{M_{1}^{2} }}{A} + \frac{{M_{2}^{2} }}{A} + \frac{{M_{3}^{2} }}{C}} \right) + F_{3} \hfill \\ \end{array} \right.$$

Combining Eq. (23) with *F*_1_ (derived above) and the fifth row in Eq. (24) yields:

29 $$\left( {\frac{\rho }{A}} \right)F_{Y} + \frac{{M_{3} }}{A}\cos \beta + \frac{{\sin^{3} \beta }}{\rho } = l.$$

Since the action line for the constraint force passes through the *Z-*axis, the following must hold:

30 $$F_{Y} = \frac{A}{\rho }\left( {l - \frac{{M_{3} }}{A}\cos \beta - \frac{{\sin^{3} \beta }}{\rho }} \right).$$

The physical meaning of this expression is that the simplified moment of the action force (*M*_*0*_) remains constant for a cross-section directed toward the *Z-*axis. The integral constant is then represented by *l* = *M*_*0*_/*A*. A combination of Eqs. (25), (26), and (30) produces:

31 $$\left\{ \begin{aligned} &F_{2} = F_{Z0} \sin \beta - \hfill \\ &\frac{A}{\rho }\left( {l - \frac{{M_{3} }}{A}\cos \beta - \frac{{\sin^{3} \beta }}{\rho }} \right)\cos \beta \hfill \\ &F_{3} = F_{Z0} \cos \beta + \hfill \\ &\frac{A}{\rho }\left( {l - \frac{{M_{3} }}{A}\cos \beta - \frac{{\sin^{3} \beta }}{\rho }} \right)\sin \beta \hfill \\ \end{aligned} \right..$$

Equations (23), (28), and (31) can be used to derive:

32 $$\left\{ \begin{aligned} & \left( {\frac{{{\text{d}}\beta }}{{{\text{d}}s}}} \right)^{2} + G\left( \beta \right) = h \hfill \\ & G\left( \beta \right) = \left[ {2(l - mcos\beta ) - \frac{{\sin^{3} \beta }}{R}} \right] \hfill \\ & \frac{\sin \beta }{R} + \frac{{2F_{Z0} }}{A}\cos \beta \hfill \\ \end{aligned} \right..$$

Equations (23), (24), and (32) produce:

33 $$F_{1} = A\left[ {h - G\left( \beta \right)} \right]^{{{\raise0.7ex\hbox{$1$} \!\mathord{\left/ {\vphantom {1 2}}\right.\kern-0pt} \!\lower0.7ex\hbox{$2$}}}} \left[ {m - \frac{3}{2R}\sin \left( {2\beta } \right)} \right].$$

In addition, an analytic expression for the normal contact force can be obtained by substituting the first rows of Eqs. (23), (31), and (33) into (24):

34 $$f = \frac{A}{{\rho^{2} }}(l - m\cos \beta - \frac{{\sin^{3} \beta }}{\rho })sin\beta + A\left[ {h - G\left( \beta \right)} \right]^{{{\raise0.7ex\hbox{${ - 1}$} \!\mathord{\left/ {\vphantom {{ - 1} 2}}\right.\kern-0pt} \!\lower0.7ex\hbox{$2$}}}} \left\{ \begin{aligned} H\left( \beta \right)\left[ {\frac{3}{2\rho }\sin \left( {2\beta } \right) - m} \right] - \hfill \\ \frac{3}{\rho }\left[ {h - G\left( \beta \right)} \right]\cos \left( {2\beta } \right) \hfill \\ \end{aligned} \right\}.$$

Here,

35 $$H\left( \beta \right) = \frac{{G^{\prime}\left( \chi \right)}}{2} = \frac{1}{\rho }(l\cos \beta - m\cos 2\beta ) - \left( {\frac{{F_{Z0} }}{A} + \frac{{2\cos \beta \sin^{2} \beta }}{{\beta^{2} }}} \right)\sin \beta .$$

From the results of Eqs. (23), (24), (31), (33), and (34), the seven unknown variables *F*_*i*_*,* (*α*, *β*, *γ*), and *f*, which define the resulting 1DFO (constrained by a cylindrical surface), can be expressed by *β* (s). Therefore, as long as the change rule for *β* (s) along the arc coordinates is satisfied, the spatial shape of the 1DFO can be determined from:

36 $$s = \int_{{\beta_{0} }}^{\beta } {\frac{d\beta }{{\sqrt {h - G(\beta )} }}} .$$

According to Eq. (23), the position coordinates for any point on a 1DFO in a fixed reference frame can be expressed as:

37 $$\left\{ \begin{array}{l} \alpha \left( s \right) = \frac{1}{\rho }\int_{0}^{s} {\sin \beta \left( \sigma \right)} d\sigma \hfill \\ Z\left( s \right) = \int_{0}^{s} {\cos \beta \left( \sigma \right)} d\sigma \hfill \\ \end{array} \right..$$

## Solution and results

### Solution algorithm design

The geometric shape of a 1DFO with cylindrical constraints was solved as follows:

1. The flexural stiffness *A*, torsional stiffness *C*, and principal moment component *M*_*i*_ (*i* = 1,2,3) were calculated for a section of the 1DFO according to given material parameters (see Eqs. (19) and (20)).
2. The constant torsion rate *ω*_30_ was calculated. Three projection values (*F*_X0_, *F*_Y0_, and *F*_Z0_) were simultaneously determined for a cross-section force at a starting point in the cylindrical coordinate system.
3. The integral constants *H, m, h, l,* and *ρ* were evaluated.
4. A second-order nonlinear differential equation with respect to *β* (s) was obtained from the previous results.
5. This differential equation can be expressed in the following standard form^38^:
  38 $$\left\{ {\begin{array}{l} {u\left( x \right)y^{\prime \prime } + v\left( x \right)y^{\prime } + w\left( x \right)y = f\left( x \right)} \\ {y\left( a \right) = \alpha ,y\left( b \right) = \beta } \\ \end{array} } \right..$$

In this paper, the theory of finite differences was used to solve Eq. (38). The specific algorithm is described as follows. The *n* equidistant discrete points of unknown function *y* (*x*) were expressed on the interval [a, b] as:

39 $$\left\{ {\begin{array}{l} {x_{k} = a + kh,k = 0,1, \cdot \cdot \cdot ,n - 1} \hfill \\ {h = \left( {b - a} \right)/\left( {n - 1} \right)} \hfill \\ \end{array} .} \right.$$

The central difference approximation was then used to replace *y″*(*x*_*k*_) and *y′*(*x*_*k*_):

40 $$\left\{ {\begin{array}{l} {y^{\prime}\left( {x_{k} } \right) \approx \frac{{y_{k + 1} - y_{k - 1} }}{2h}} \\ {y^{\prime\prime}\left( {x_{k} } \right) \approx \frac{{y_{k + 1} - 2y_{k} + y_{k - 1} }}{{h^{2} }}} \\ \end{array} } \right..$$

A numerical iteration of the original differential equation could then be expressed as follows:

41 $$\left\{ {\begin{array}{*{20}l} {\frac{{u\left( {x_{k} } \right)}}{{h^{2} }}\left( {y_{{k + 1}} - 2y_{k} + y_{{k - 1}} } \right) + } \\ {\frac{{v\left( {x_{k} } \right)}}{{2h}}\left( {y_{{k + 1}} - y_{{k - 1}} } \right) + w\left( {x_{k} } \right)y_{k} = f\left( {x_{k} } \right)} \\ {k = 0,1, \cdot \cdot \cdot ,n - 1} \\ \end{array} } \right.$$

The following equations for *y*_*k*_ were then obtained:

42 $$\left\{ {\begin{array}{*{20}l} {y_{0} = \alpha } \\ {p_{k} y_{{k - 1}} + q_{k} y_{k} + r_{k} y_{{k + 1}} d_{k} .} \\ {y_{{n - 1}} = \beta } \\ \end{array} } \right.$$

Here,

43 $$\left[ {\begin{array}{*{20}c} {q_{0} } & {r_{0} } & {} & {} & {} \\ {p_{1} } & {q_{1} } & {r_{1} } & {} & {} \\ {} & \ddots & \ddots & \ddots & {} \\ {} & {} & {p_{n - 2} } & {q_{n - 2} } & {r_{n - 2} } \\ {} & {} & {} & {p_{n - 1} } & {q_{n - 1} } \\ \end{array} } \right]\left[ {\begin{array}{*{20}l} {y_{0} } \\ {y_{1} } \\ \vdots \\ {y_{n - 2} } \\ {y_{n - 1} } \\ \end{array} } \right] = \left[ {\begin{array}{*{20}l} {d_{0} } \\ {d_{1} } \\ \vdots \\ {d_{n - 2} } \\ {d_{n - 1} } \\ \end{array} } \right],$$

and

44 $$\begin{array}{*{20}c} {q_{0} = 1,r_{0} = 0,d_{0} = \alpha ;} \hfill \\ {p_{n - 1} = 0,q_{n - 1} = 1,d_{n - 1} = \beta .} \hfill \\ \end{array}$$

**Figure 5:**
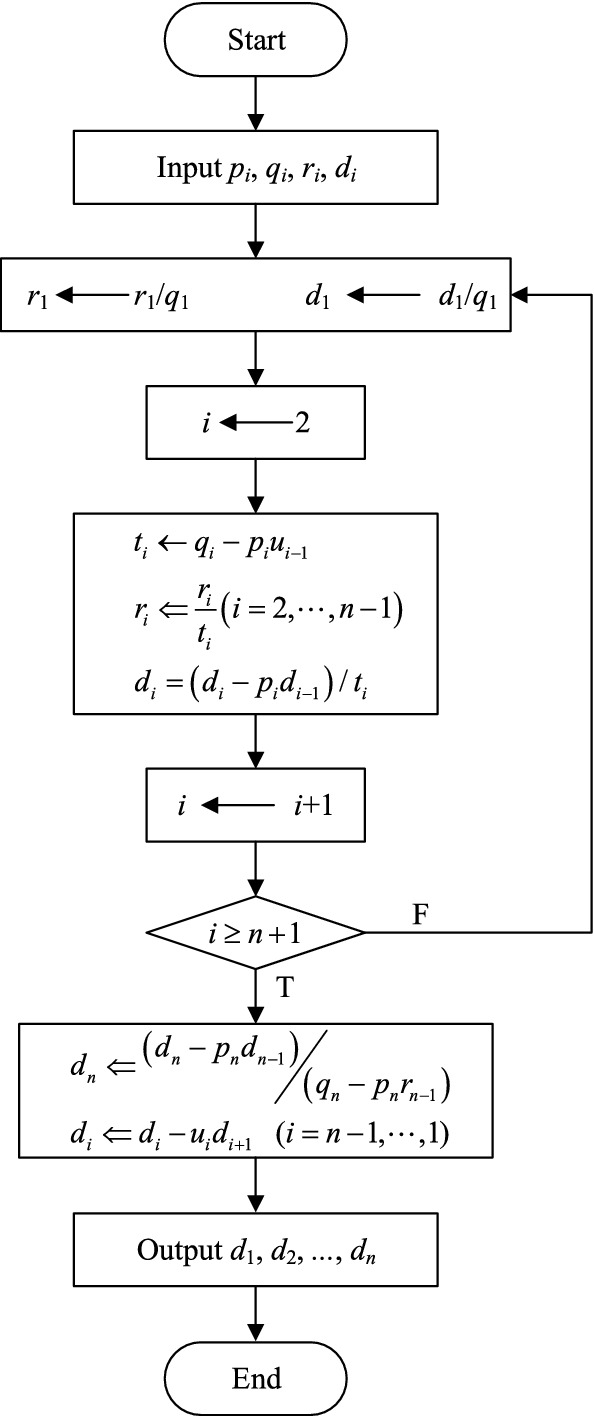
A flowchart for the proposed method.

Equation (43) is a tridiagonal system of equations, which can be solved with the pursuit method^39^. A flowchart for this algorithm is shown in Fig. 5.

In order to improve the accuracy of practical calculations, the following improvement measures were adopted:

1. A set of approximate solutions at *n − 2* equidistant discrete points were obtained by calculating the step length *h* = *(b − a)/(n − 1)*:
  45 $$y_{k}^{h} = y_{k} (a + kh),\quad k = 1,2, \cdots ,n - 2.$$
2. Another group of approximate solutions at *n − 2* equidistant discrete points were obtained by calculating the step length *h/2* = *(b − a)/(2n − 2)*:
  46 $$y_{k}^{h/2} = y_{k} (a + kh),\quad k = 1,2, \cdots ,n - 2.$$
3. Finally, the iteration format was as follows^41^:
  47 $$y_{k} = \frac{{4y_{k}^{h/2} - y_{k}^{h} }}{3},\quad k = 1,2, \cdots ,n - 2.$$

This approach constitutes the novel hybrid semi-analytic method developed for this study.

### Geometric shape simulation in an equilibrium state

In this paper, a geometric shape simulation was developed for a 1DFO using the Visual Studio environment, based on an open geometric kernel source system named Open Cascade (OCC).

In addition to the basic functions of three-dimensional graphics display and operation, the most prominent function of the developed platform was its ability to render three-dimensional shapes and save output in the general CAD format (which can be read by any 3D CAD software). The basic approach to 1DFO shape simulation is to first calculate a centerline and cross-section attitude using given boundary and initial conditions. Adjacent nodes are then interpolated with a B-spline curve to obtain the centerline shape. A scanning envelope is then implemented along the centerline using a circle with a radius equal to that of the cross-section, producing the final three-dimensional 1DFO model^40^.

A model 1DFO with a length of *L* = 20 cm was developed for testing purposes. The material parameters, initial Euler angles, and constraint boundary conditions are described below.

**Figure 6:**
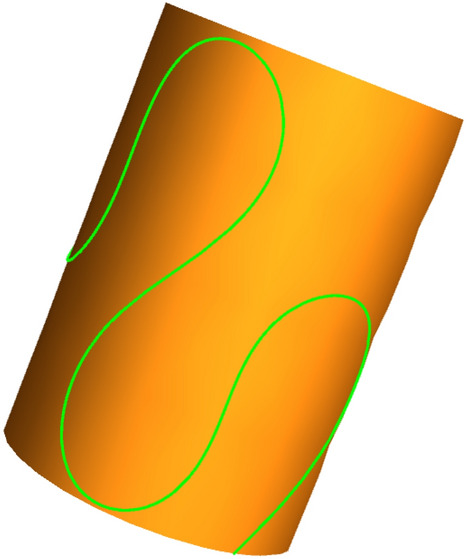
The geometric shape of the 1DFO constrained by a cylindrical surface.

The geometric shape of a 1DFO under these condition is shown in Fig. 6. From the figure, it is evident that even under the assumption of ideal contact constraints, 1DFOs with cylindrical constraints exhibit complex geometric structures.

**Figure 7:**
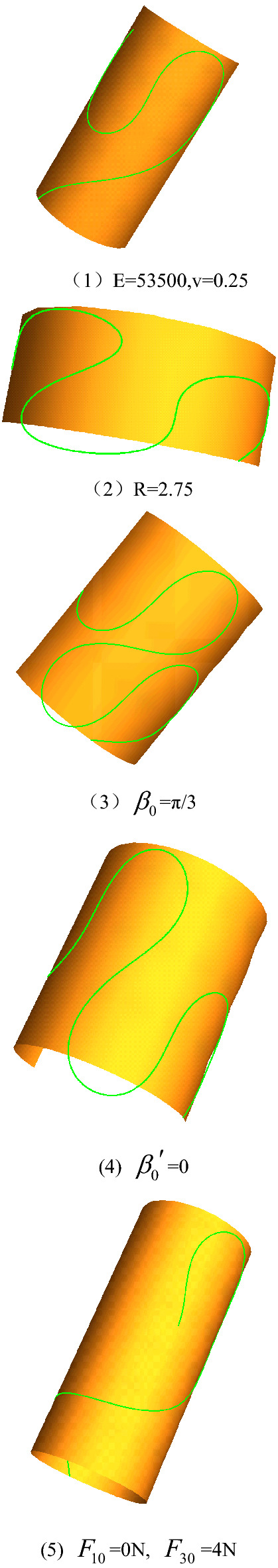
The geometric shape of the 1DFO constrained by a cylindrical surface under various geometric boundary conditions.

To further illustrate the validity of the model proposed in this paper, a shape simulation was conducted by varying the material parameters, Euler angles and, boundary constraint forces. The results are shown in Fig. 7.

In (1), with the increase of elastic modulus, the number of bending of 1DFO decreases, making it less prone to bending. When the cylinder radius increases, 1DFO attached to the cylinder surface in (2) has a smaller bending amplitude. In (3) and (4), as the initial Euler Angle and the terminal Euler Angle increase, the number and degree of bending of 1DFO increase. In (5), when the contact force is reduced, the attachment degree of 1DFO on the cylinder becomes smaller. To sum up, these body shape changes are in line with the law of reality. So the modeling and solving methods proposed are accurate in this paper.

These numerical calculations showed that a 1DFO constrained by a cylindrical surface generally exhibits a complex geometry. The shape of this 1DFO changed when the material constants, geometric characteristics, external constraints, or boundary conditions changed. The effects of external constraints and boundary conditions appeared to be larger than that of the physical constants or geometry. The pure torsion of the cross-section relative to the Frenet coordinate system is not reflected in the figures.

## Conclusion

In this study, a novel modeling and geometric simulation method, based on Cosserat elastic rod theory, was proposed for surface-constrained 1DFOs. Potential applications include DNA molecules wrapped around proteins and drill pipes in contact with borehole walls or submarine cables. Specific conclusions were as follows.

1. Surface constraints on the position and shape of the 1DFO were described by two constant geometric constraint equations. As such, four generalized coordinates were needed to describe the position and shape of the 1DFO cross-section. Including three force vector components, the equilibrium equation for the geometric cross-section was composed of seven independent equations.
2. Using a 1DFO constrained by a cylindrical surface as an example, a novel hybrid semi-analytical numerical method was proposed to resolve the second-order nonlinearity of the equations. A Hamiltonian function and an initial integral operator were introduced in cylindrical coordinates to derive the analytical solution in decoupled polar coordinates. This approach avoids the poor stability and efficiency of general numerical techniques such as Euler's method.
3. The analytic solution cannot be expressed by this function in its implicit form. Therefore, an improved pursuit method was introduced to solve this problem, ensuring the accuracy and efficiency of the numerical calculation. The geometric shape of the 1DFO with cylindrical constraints under various boundary conditions was simulated using the shape simulation platform developed by an open source geometric kernel system.
4. Numerical simulations showed that the effects of external and boundary constraints at both ends of the 1DFO were greater than that of physical constants or the geometric characteristics of the cylinders.

These results suggest the model and algorithm proposed in this paper can effectively simulate 1DFO surfaces. This approach could be a valuable new tool for the mechanical analysis of elongated structures, such as DNA molecules, drill pipes, and submarine cables. In the future, this model will be expanded to include 1DFOs in a viscous medium.

## Data Availability

All data generated or analyzed during this study are included in this published article.
